# Tuberculosis in a Migrant Population: Integrated Management of a Case through the Prevention Department and Hospital Services

**DOI:** 10.3390/microorganisms12061216

**Published:** 2024-06-17

**Authors:** Nahuel Fiorito, Daniela Piacentini, Serena Cian, Anna Voltolini, Jacopo Fagherazzi, Erica Bino, Marika Brancher, Giorgia De Luca, Marica Battistin, Mattia Manzi, Vincenzo Marcotrigiano, Angela Vedana, Christian Napoli, Sandro Cinquetti

**Affiliations:** 1 Prevention Department, Local Health Authority “ULSS 1 Dolomiti”, 32100 Belluno, Italy; nahuel.fiorito@aulss1.veneto.it (N.F.); serena.cian@aulss1.veneto.it (S.C.); anna.voltolini@aulss1.veneto.it (A.V.); jacopo.fagherazzi@aulss1.veneto.it (J.F.); erica.bino@aulss1.veneto.it (E.B.); marika.brancher@aulss1.veneto.it (M.B.); giorgia.deluca@aulss1.veneto.it (G.D.L.); marica.battistin@aulss1.veneto.it (M.B.); mattia.manzi@aulss1.veneto.it (M.M.); sandro.cinquetti@aulss1.veneto.it (S.C.); 2 Infectious Diseases Unit, Specialistic Area Department, Local Health Authority “ULSS 1 Dolomiti”, 32100 Belluno, Italy; daniela.piacentini@aulss1.veneto.it; 3 Department of Statistical Sciences, University of Padova, 35122 Padova, Italy; angela.vedana@studenti.unipd.it; 4 Department of Medical Surgical Sciences and Translational Medicine, Sapienza University of Rome, 00185 Rome, Italy; christian.napoli@uniroma1.it; 5 National Institute for Health, Migration and Poverty, 00153 Rome, Italy

**Keywords:** tuberculosis, migrant, public health, clinical management, healthcare workers

## Abstract

Among numerous public health actions, the Prevention Departments of Local Health Authorities take charge of the migrant asylum-seeking population for health assessments, for the implementation of preventive activities, and for any consequent actions. This report describes two cases of tuberculosis in Belluno Province managed by a multidisciplinary team made up of healthcare workers that involved numerous diagnostic, clinical, and prophylactic implications, as well as an analysis of the epidemiological aspects related to the incidence of cases along the migration route. Although the cases occurred in a northeastern Italian territory, the management methods described here may represent good practices to share on this operational line, which can promote the strengthening of cooperation between Health Authorities and Emergency Reception Centers to correctly identify cases of active tuberculosis that may not have been initially screen-detected.

## 1. Overview

Tuberculosis is a contagious infectious disease caused by a bacterium, *Mycobacterium tuberculosis* (*M. tuberculosis*) [1]. Approximately 25% of the world’s population is infected with *M. tuberculosis*, but only a small proportion (approximately 10%) develop the disease during their lifetime [2]. In 10% of people, the bacterium is eliminated from the body; in the remaining 90%, a latent tuberculosis infection develops [3]. The risk of experiencing clinical disease has been estimated at 5% during the first year after infection and a further 5% throughout life [4].

Infection occurs through the inhalation of droplets/particles 1–5 µm in diameter containing *M. tuberculosis*, which are expelled from patients with active pulmonary tuberculosis, typically when they cough [3]. *M. tuberculosis* can remain in an inactive state for a long time [5,6,7]. Some infected individuals transition from a state of latent tuberculosis infection to a state of active infection [6,8,9].

People with latent tuberculosis infections have no symptoms and are not contagious. The clinical form can be pulmonary or extrapulmonary [4,10].

A diagnosis of tuberculosis is made based on the isolation of *M. tuberculosis* from body fluids such as sputum, bronchoaspiration, pleural fluid, CSF, blood, or from tissues [11]. Additional diagnostic tools include direct microscopic analysis of the sample to search for alcohol acid-fast bacilli and the use of molecular nucleic acid amplification tests. In addition, immunological tests can be performed (tuberculin reactions and/or IGRAs), but the positive results of which may only represent previous exposure to the mycobacterium and do not necessarily correlate with active disease [12].

Tools for diagnosing tuberculosis also include radiographic imaging, specifically chest X-ray, which is the initial approach to the diagnostic evaluation of patients with suspected tuberculosis. Computed tomography is often useful due to its greater sensitivity [4,13].

The traditional treatment of a patient with a lung infection in the absence of drug resistance includes a four-drug induction phase (rifampicin, isoniazid, pyrazinamide, and ethambutol) lasting 2 months, followed by a two-drug maintenance phase (rifampicin and isoniazid) for a minimum of 4 months, always in association with pyridoxine supplementation to reduce the risk of neuropathy associated with taking isoniazid. However, treatment will need to be modulated based on the results of the antibiogram [14].

A multidisciplinary tuberculosis team should implement strategies to encourage patients to stay on treatment and prevent early treatment discontinuation. It is advisable to carry out a risk assessment of all patients with tuberculosis disease to identify their needs and, if necessary, strengthen their case management (e.g., through a personalized therapeutic package that includes using supportive actions such as directly observed therapy (DOT), educating patients on tuberculosis and its treatment, etc.) [15].

The Global Tuberculosis Report 2022 notes that in 2021, tuberculosis was one of the leading causes of death from a single infectious agent. Globally, the COVID-19 pandemic hindered progress on controlling tuberculosis and achieving global targets. The most obvious impact was sharp declines in the numbers of people newly diagnosed with tuberculosis, falling from a peak of 7.1 million in 2019 to 5.8 million in 2020 (−18%) and 6.4 million in 2021. According to the joint ECDC and WHO Europe document “Tuberculosis surveillance and monitoring in Europe 2023–2021 data”, it is estimated that in the WHO European Region in 2021, 230,000 people fell ill with tuberculosis (25 cases per 100,000 people).

Although the European Region achieved the important objective (set by the end TB strategy) of a 20% cumulative reduction in the incidence of tuberculosis for the period 2015–2020, in 2021, for the first time in two decades, the estimated incidence rate increased by 1.2% compared to 2020. This turnaround reflects the impact of the disruption to tuberculosis health services caused by the COVID-19 pandemic.

In 2021, in the EU and EEA member states, 33,520 cases of tuberculosis were reported (7.3 cases per 100,000 inhabitants), with Romania accounting for 23.8% of the total with 41.6 cases per 100,000 inhabitants.

The overall notification rates confirm the downward trend from 2012 to 2021, with a decrease of 45% and an average annual decrease of between 6.9 and 10.3% in all age groups. Again, the control measures put in place for the COVID-19 pandemic may have had an impact on surveillance and services dedicated to tuberculosis.

The data for Italy from the tuberculosis case notification system of the Ministry of Health and published in “Tuberculosis surveillance and monitoring in Europe 2023”, which refers to the data for the year 2021, confirmed that Italy is among the countries with a low incidence of disease (<20/100,000). In 2021, 2480 cases of tuberculosis were reported, corresponding to a notification rate of 4.0 cases per 100,000 inhabitants, a slight increase compared to the previous year (2287 cases, incidence of 3.8/100,000). Overall, 57.9% of cases (N = 1437) were reported in people of foreign origin.

Overall, 1686 cases (68.0%) were classified as new diagnoses in people never previously treated for tuberculosis [16].

The aim of this case report is to provide an overview of the management of tuberculosis positivity detected after initial negative screening, with the related public health implications.

## 2. Internal Management of Tuberculosis Contacts

Active research on and contact monitoring of cases of pulmonary tuberculosis are two of the most important preventive measures to implement appropriate public health actions to control the disease [17]. Therefore, it is essential to evaluate the type of contacts involved in the case, and contacts can be classified into three categories:

- Close contacts: people who live with the patient or who have shared the same confined space for numerous hours a day;
- Regular contacts: people who regularly share the same closed space;
- Occasional contacts: people who occasionally share the same closed place.

The screening principle involves proceeding through concentric circles around the patient. Screening must start with people who have had close contact and, where appropriate, people who request it spontaneously. If investigations of close contacts rule out transmission, the investigation can be limited to this group. Otherwise, regular contacts must be examined, and similarly, if there is evidence of transmission of infection between subjects in this second category, the investigation must be extended to occasional contacts. According to the national protocol, the diagnosis tests available in our laboratory hospital, and in the entire region, are represented by tuberculin skin test (TST) and IGRAs [18,19]. The IGRA test shows good promise for improving the TB diagnosis in immunocompetent children aged > 5 years, coming from high-income settings, while in immunocompromised children and in developing countries, IGRAs’ performance is equivalent or inferior to TST [20].

The TST has been used for tuberculosis screening; however, its low specificity among BCG-vaccinated healthcare workers (HCWs) and the boosting phenomenon of repeated TSTs can lead to false positive results, with the potential negative consequence of unnecessary chest X-rays and/or isoniazid prophylaxis. In vitro interferon-gamma release assays (IGRAs) have demonstrated superior specificity and sensitivity compared with TSTs and require only one visit [21]. Furthermore, the current national guidelines for the control of tuberculosis among immigrants in Italy reflect the contents of a systematic review performed by Nienhaus et al. on the cost-effectiveness of screening for latent tuberculosis infection (LTBI) in high-risk groups, showing strong evidence in favor of using IGRA alone compared to TST or the combination of TST and IGRA. According to a cost-effectiveness analysis, the higher unit cost of IGRA is compensated by a more rational use of chest X-rays and LTBI chemoprophylaxis following the use of this test [22].

Therefore, given the high incidence of tuberculosis in countries of origin linked to investigated cases (Table 1), it was agreed to adopt, despite the greater economic impact, the quantiferon test as a first screening.

**Figure 1:**
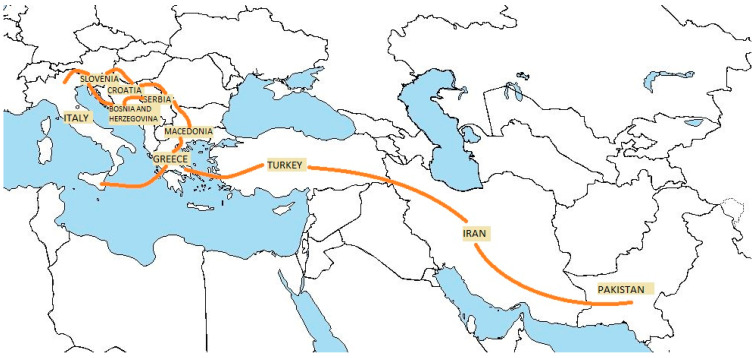
Map of migrants’ route related to countries crossed.

The cases of tuberculosis described in this case report involve people who arrived in our territory following the route illustrated in the map (Figure 1) [23]. At the initial screening, coinciding with the first access to the Hygiene and Public Health Service (SISP), tacit informed consent for the use and processing of personal data is requested, including for study purposes. Subsequently, each asylum seeker is issued a Temporarily Present Foreigner (STP) card, a community admission certificate, and possibly a vaccination certificate. Table 1 reports the incidence of tuberculosis as of February 2024 in the countries where such people may have traveled [24].

## 3. Case Description

On 20 October 2023, an Emergency Reception Center (CAS) operating in Belluno Province contacted the Prevention Department of Local Health Authority ULSS 1 Dolomiti to report the finding of a positive quantiferon test for one of their guests.

The subject (case 1), a 32-year-old male from Pakistan benefiting from a residency permit, had arrived in Belluno in February 2023. During his first health screening, he appeared to be in good health; serological tests were negative for HBV, HCV, and HIV; syphilis and quantiferon tests were also negative.

Nevertheless, he had been experiencing asthenia, weight loss, cough, fever, chest pain, and lymphadenopathy since the end of August, and considering the diagnostic suspicion, he was being taken care of by his general practitioner. To manage the case, the Infectious Diseases Unit of Belluno Hospital was contacted, and a dedicated multidisciplinary team was set up.

**Figure 2:**
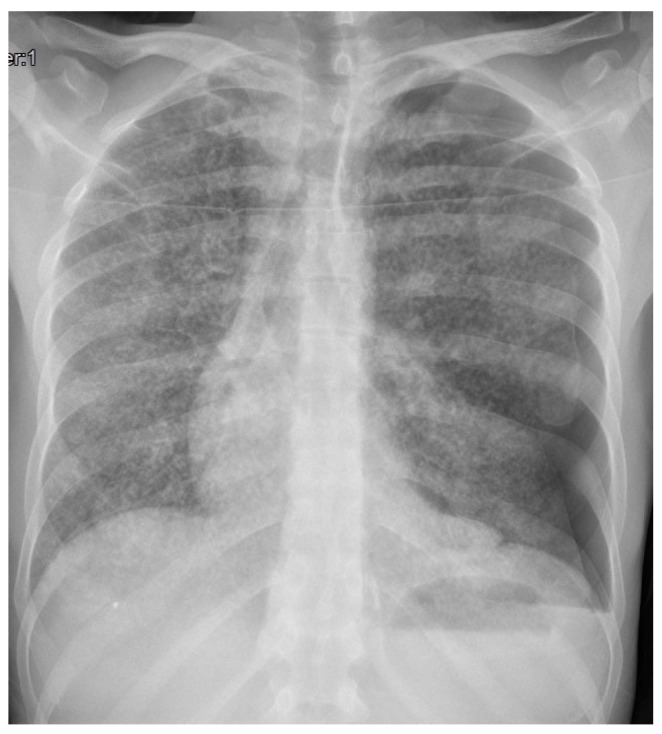
Chest X-ray of case n. 1.

The patient was immediately referred to the hospital’s emergency department to perform the first tests. The chest X-ray showed a pattern suggestive of miliary tubercular localization, as shown in Figure 2 (case 1), and he was admitted to the Infectious Diseases Unit on the same day, then discharged after about a month (23 November 2023).

While awaiting further diagnostic analysis and assessment of contagiousness, the SISP started an epidemiological investigation, which revealed close contact with another asylum seeker, who was being investigated for suspected symptoms and a positive quantiferon test in August 2023. Unfortunately, at the time of the investigation, he was no longer a guest of the CAS as he had suddenly left the center. However, even with an extended time scale and logistical problems due to the difficult availability of the subject, thanks to the collaboration of CAS operators and the hospital’s multidisciplinary team, he continued to be checked for tuberculosis; there was a suggestive radiological picture but a negative bronchoaspirate culture.

The following close contacts were identified from the investigation: seven CAS workers and nine cohabitants (including two roommates), who were scheduled for screening as required by the protocol. The seven CAS operators tested negative in the first screening. Among the cohabitants, one, who was already being followed by the Infectious Diseases Unit for previous LTBI, underwent a further chest X-ray, which was negative. Seven cohabitants tested negative on the quantiferon test, while one tested positive (case 2).

On 23 October 2023, the first patient (case 1) underwent bronchoaspiration, and in the following days, the laboratory analysis confirmed the contagiousness of the case based on a positive test for mycobacterium TBC (DNA).

The second patient (case 2), a 23-year-old male who had arrived in Italy from Pakistan in December 2022, appeared to be in good health and had a negative quantiferon test during the first health screening.

**Figure 3:**
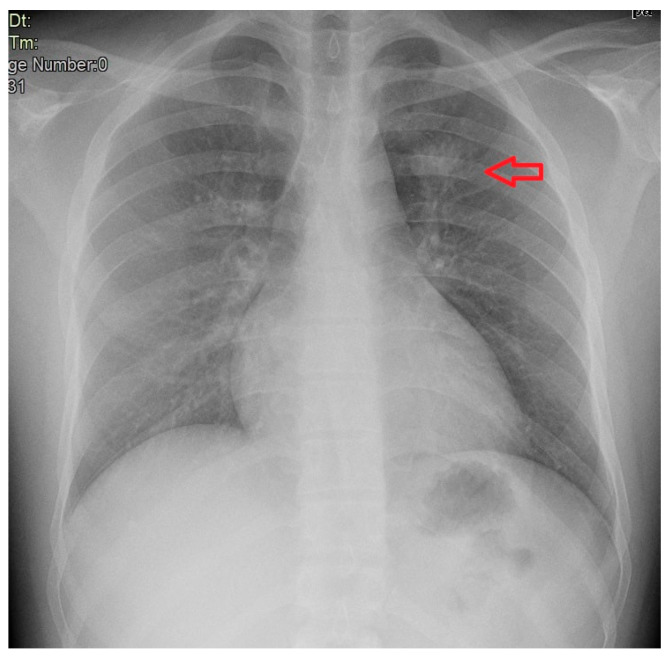
Chest X-ray of case n. 2.

Following the positive quantiferon screening test, diagnostic investigations were carried out, including a chest X-ray, showing a parenchymal consolidation, as shown in Figure 3 (case 2), and subsequent hospitalization in the Infectious Diseases Unit from 26 October 2023 to 4 January 2024.

Hence, following the positivity of case 2 and as a precaution, the six contacts of case 1, despite their negative quantiferon tests, underwent chest X-rays, which were negative.

SISP HCWs continued the previously launched epidemiological investigation by evaluating the new contacts. The investigation revealed that the second patient (case 2) had shared a room with the first patient (case 1) for 7 months and for a few months had been living in another house made up of two apartments whose 19 inhabitants were all asymptomatic. Among them, the following was found:

- One subject who recently underwent a health screening and tested positive in the quantiferon test but had a negative X-ray was already in the care of the Infectious Diseases Unit;
- Three subjects who had a positive quantiferon test in the past and had already finished therapy for LTBI underwent a control X-ray, which was negative;
- Thirteen subjects had negative quantiferon tests on 9 November 2023;
- Two subjects who underwent a quantiferon test on 9 November 2023 resulting in minimal signs of reactivity (low-intensity positivity) had negative chest X-rays. They were taken over by Infectious Diseases to start therapy for LTBI.

On 3 November 2023, the analysis laboratory confirmed the positivity of case 2, based on the presence of rare alcohol acid-resistant bacilli in the bronchoaspirate.

As a precaution, having confirmed the positivity of the subject, the CAS, in agreement with the prefecture, suspended the reception of further asylum seekers until 8 November 2023, when the director of the Prevention Department authorized the restoration of normal activity.

On 12 December 2023, one month later, the two contacts whose status was previously found to be doubtful, because of low-intensity positivity of TB antigen tube TB2 and negativity of TB antigen tube TB1 (which has reportedly been associated with recent *M. tuberculosis* infection), again underwent quantiferon tests, and one was still doubtful while one was positive [25,26].

The positive contact was sent for a new chest X-ray with an ultrasound of the abdomen. This contact, no longer a guest of the CAS, who tested negative on the culture test, underwent a repeat chest CT scan 6 months later.

The contacts of case 1, whose first screening test was negative, were recalled for a second screening 2 months later, at the beginning of January 2024, per protocol, and all still tested negative. The contacts of case 2, following the first negative screening, were contacted for a second screening in mid-January; 11 were negative, 2 doubtful, and 1 positive. The positive contact was sent for a chest X-ray, which was negative, and was taken in for care by the Infectious Diseases Unit for LTBI.

The two contacts who were doubtful underwent quantiferon tests after a month, which were positive, with negative chest X-rays, and they were subsequently referred to the Infectious Diseases Unit for LTBI.

From the investigation conducted by SISP, the following situation emerged:

- Two positive cases were under treatment in the Infectious Diseases Unit;
- Seven CAS operators had two negative screening tests;
- One subject, who was no longer a guest of the CAS, underwent diagnostic tests;
- Eighteen CAS contacts tested negative on both screening tests;
- Nine CAS contacts were diagnosed with LTBI.

**Figure 4:**
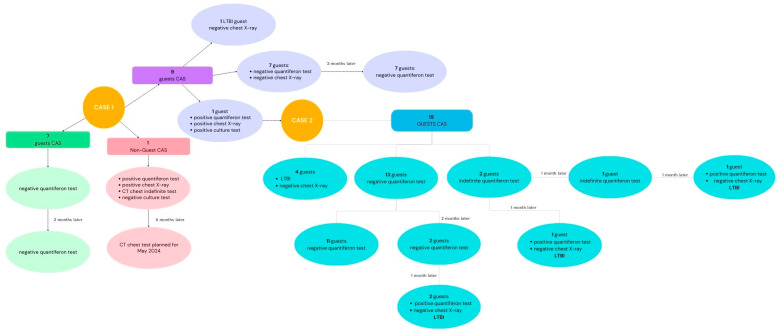
Flowchart of the cases described.

The graphic evidence regarding the results from the contact tracing of the case is described in a flowchart in Figure 4.

## 4. Clinical Case Management

On admission to the Infectious Diseases Unit, the first patient presented with compromised general conditions and cachexia. His physical examination revealed a body temperature of 37.6 °C, an SpO2 of 96%, a blood pressure of 112/71 mmHg, and a heart rate of 110 beats/min. Laboratory tests revealed a normal leukocyte count (5070 cells/μL) with lymphopenia (360 cells/μL) and a slightly elevated serum level of C-reactive protein (1.78 mg/dL); hepato-renal values were normal, but hypoalbuminemia (3 g/dL) and marked hyponatremia (129 mmol/L) were found. The HIV screening test was negative. The chest X-ray showed diffuse reticular nodular thickening compatible with miliary tuberculosis and extended left pneumothorax, with the need for positioning of thoracostomy drainage (Figure 2).

Due to the patient’s inability to produce sputum samples, a bronchoscopy was performed. Rare acid-fast bacilli were found on the bronchoalveolar lavage sample by multiplex real-time PCR assay, identifying *M. tuberculosis* complex. This result was confirmed by means of culture. Blood samples were negative for mycobacteria, and no extrapulmonary localizations were found.

**Figure 5:**
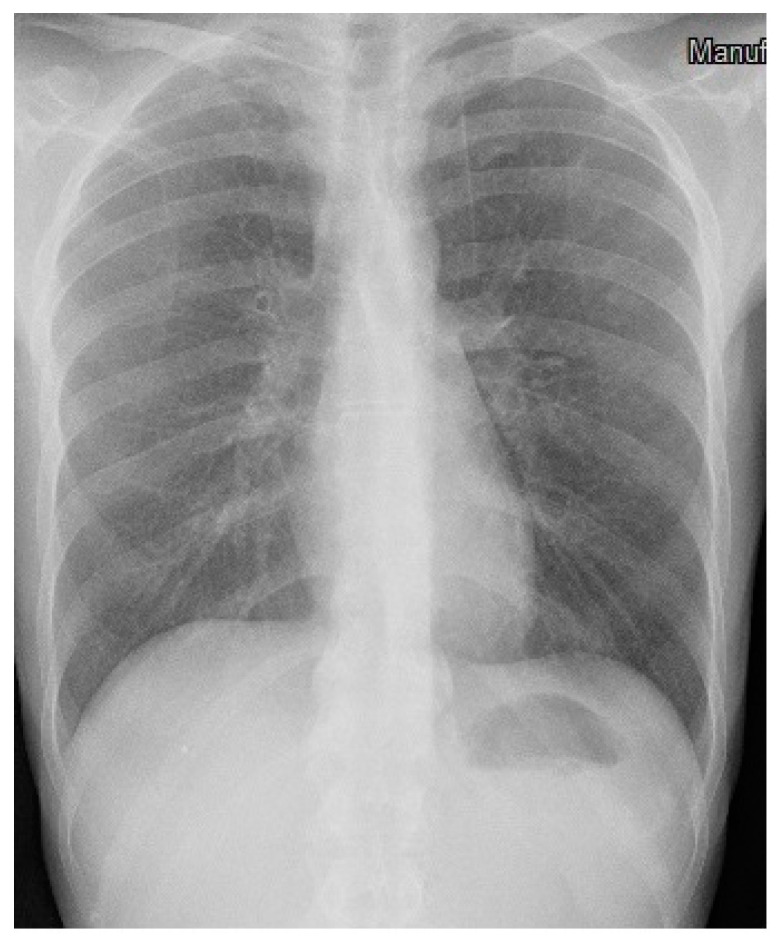
Follow-up chest X-ray of case n. 1.

The patient was started on multiple anti-TB drugs, including isoniazid, rifampicin, ethambutol, and pyrazinamide, and had a negative bronchoalveolar lavage for acid-fast bacilli after 1 month of treatment. A control chest X-ray showed a recurrence of pneumothorax, which was conservatively treated and was resolved at the 3-month checkup (Figure 5).

After a month of hospitalization, the patient was discharged and started on outpatient management. At the next examination, he was found to be in poor compliance with the treatment and was hospitalized again to resume antitubercular therapy and arrange for domiciliary DOT.

The second patient was admitted a week after the first one to exclude pulmonary tuberculosis. He presented with a poorly productive cough in the absence of other associated symptomatology. Laboratory tests revealed a normal leukocyte count (6130 cells/μL) with normal serum levels of C-reactive protein and negative serology for HIV. Chest X-ray showed a pulmonary consolidation in the left upper lobe (Figure 3). Also in this case, rare acid-fast bacilli were found in the bronchoalveolar lavage sample, with the presence of *M. tuberculosis* confirmed by culture. The standard four-drug anti-TB therapy was started, but the bronchoalveolar lavage sample took 2 months to become negative. The patient was then discharged with a two-drug regimen of isoniazid and rifampicin. During the hospital stay, the patient also received treatment for scabies.

Both patients are still on treatment with regular outpatient checkups. Adherence to drug treatment is guaranteed by domiciliary DOT.

## 5. Conclusions

The outcomes of this investigation highlight the importance of proper tuberculosis screening among the migrant population arriving in Italy and epidemiological analysis of their countries of origin and those traveled through during their migration.

This study reveals that is possible to develop the disease following an initial negative screening, likely due to the presence of contagion during the journey, and that infection can potentially spread within reception facilities, even small ones, before the second screening is performed.

The positive results of bronchoalveolar lavage cultures in both cases over a very short time interval did not allow for unequivocal identification of the primary case. Furthermore, the presence of a patient with evidence of symptomatic tuberculosis, albeit non-bacillary, highlights the criticality of managing migrants with suspected cases once they leave the reception facility, which can lead to extended timelines for investigations and therapeutic management.

Among the strengths of the case investigated are the synergistic operation of the hospital and territorial network, in both taking charge of and managing the case, and the multiplicity of HCWs specifically involved in these operational lines [27,28].

For these reasons, it is considered important to strengthen the cooperation between Health Authorities, reception centers, and general practitioners for the early identification of any cases of active tuberculosis that are not detected during initial screening, paying attention to symptoms that emerge in this population during their stay in CAS and after they leave.

## Figures and Tables

**Table 1:** Estimated incidence of tuberculosis in countries where migrants may have traveled, year 2021.

Countries, Territories, and Areas	Number of Incident Tuberculosis Cases (C.I. 95%)	Incidence of Tuberculosis * (C.I. 95%)
Pakistan	615,000 (450,000–821,000)	266 (194–355)
Iran	10,000 (7800–13,000)	12 (8.8–15)
Turkiye	12,000 (9600–15,000)	14 (11–18)
Greece	220 (190–260)	2.1 (1.8–2.5)
North Macedonia	240 (180–300)	11 (8.4–14)
Serbia	1100 (900–1200)	14 (12–17)
Bosnia and Herzegovina	810 (610–1000)	25 (19–32)
Croatia	180 (150–200)	4.4 (3.7–5)
Slovenia	91 (78–100)	4.3 (3.7–5)
Italy	2700 (2300–3200)	4.6 (3.9–5.3)

* per 100,000 population per year.

## Data Availability

Data are contained within the article.
